# Health care and societal costs of the management of children and adolescents with attention-deficit/hyperactivity disorder in Spain: a descriptive analysis

**DOI:** 10.1186/s12888-017-1581-y

**Published:** 2018-02-08

**Authors:** Javier Quintero, Josep A. Ramos-Quiroga, Javier San Sebastián, Francisco Montañés, Alberto Fernández-Jaén, José Martínez-Raga, Marta García Giral, Montserrat Graell, María J. Mardomingo, César Soutullo, Jesús Eiris, Montserrat Téllez, Montserrat Pamias, Javier Correas, Juncal Sabaté, Laura García-Orti, José A. Alda

**Affiliations:** 1 Psychiatry Department, Hospital Universitario Infanta Leonor and Psychiatry and Medical Psychology Department, Complutense University, Madrid, Spain; 2 Department of Psychiatry, Hospital Universitari Vall d’Hebron, Barcelona, Spain; Biomedical Network Research Centre on Mental Health (CIBERSAM), Barcelona, Spain; and Department of Psychiatry and Legal Medicine, Universitat Autònoma de Barcelona, Bellaterra, Spain; 3 Child and Adolescent Psychiatry Department, Hospital Universitario Ramón y Cajal, Madrid, Spain and Psychiatry Department, Universidad de Alcalá, Madrid, Spain; 4 Hospital Universitario Fundación de Alcorcón, Madrid, Spain and Rey Juan Carlos I University, Madrid, Spain; 5 Department of Neuropediatrics, Hospital Universitario Quirón, Madrid, Spain; 6 UDPyPC, Hospital Universitario Dr. Peset and University of Valencia and University CEU-UCH, Valencia, Spain; 7 Child and Adolescent Psychiatry and Psychology Department, Institute of Neuroscience, Hospital Clinic, Barcelona, Spain; 8 Child and Adolescent Psychiatry and Psychology Department, Hospital Infantil Universitario Niño Jesús and Biomedical Network Research Centre on Mental Health (CIBERSAM), Madrid, Spain; 9 Child and Adolescent Psychiatry Department, Hospital Gregorio Marañón, Universidad Complutense, Madrid, Spain; 10 Child and Adolescent Psychiatry Unit, Department of Psychiatry and Medical Psychology, University of Navarra Clinic, Pamplona, Spain; 11 Neuropediatric Division, Pediatric Department, Complejo Hospitalario Universitario Santiago De Compostela, Santiago de Compostela, Spain; 12 Department of Neuropediatrics, Hospital La Fe, Valencia, Spain; 13 Hospital Parc Taulí, Sabadell, Spain and Autonomous University of Barcelona, Bellaterra, Spain; 14 Psychiatry and Mental Health Department, Hospital Universitario del Henares, Francisco de Victoria University, Madrid, Spain; 15 Shire, Madrid, Spain; 16 Child and Adolescent Psychiatry and Psychology Department, Hospital Sant Joan de Déu, Esplugues de Llobregat, 08950 Barcelona, Spain

**Keywords:** ADHD, Adolescence, School children, Economic evaluation

## Abstract

**Background:**

Attention-deficit/hyperactivity disorder (ADHD) is a common neurodevelopmental condition in childhood (5.3% to 7.1% worldwide prevalence), with substantial overall financial burden to children/adolescents, their families, and society. The aims of this study were to describe the clinical characteristics of children and adolescents with ADHD in Spain, estimate the associated direct/indirect costs of the disorder, and assess whether the characteristics and financial costs differed between children/adolescents adequately responding to currently available pharmacotherapies compared with children/adolescents for whom pharmacotherapies failed.

**Methods:**

This was a multicenter, cross-sectional, descriptive analysis conducted in 15 health units representative of the overall Spanish population. Data on demographic characteristics, socio-occupational status, social relationships, clinical variables of the disease, and pharmacological and non-pharmacological treatments received were collected in 321 children and adolescents with ADHD. Direct and indirect costs were estimated over one year from both a health care system and a societal perspective.

**Results:**

The estimated average cost of ADHD per year per child/adolescent was €5733 in 2012 prices; direct costs accounted for 60.2% of the total costs (€3450). Support from a psychologist/educational psychologist represented 45.2% of direct costs and 27.2% of total costs. Pharmacotherapy accounted for 25.8% of direct costs and 15.5% of total costs. Among indirect costs (€2283), 65.2% was due to caregiver expenses. The total annual costs were significantly higher for children/adolescents who responded poorly to pharmacological treatment (€7654 versus €5517; *P* = 0.024), the difference being mainly due to significantly higher direct costs, particularly with larger expenses for non-pharmacological treatment (*P* = 0.012).

**Conclusions:**

ADHD has a significant personal, familial, and financial impact on the Spanish health system and society. Successful pharmacological intervention was associated with lower overall expenses in the management of the disorder.

**Electronic supplementary material:**

The online version of this article (10.1186/s12888-017-1581-y) contains supplementary material, which is available to authorized users.

## Background

Attention-deficit/hyperactivity disorder (ADHD) is a common neurodevelopmental condition in childhood, with an estimated worldwide prevalence in children/adolescents ranging from 5.3% to 7.1%, with little variation across countries and regions [1–3]. ADHD is characterized by developmentally inappropriate levels of inattention, hyperactivity, or impulsivity that have a significant impact on different aspects of the child/adolescent’s everyday life, including social, academic, or occupational activities. The severity of ADHD and presence of particular symptoms is highly heterogeneous, and the Diagnostic and Statistical Manual of Mental Disorders Fifth Edition (DSM-5) distinguishes clinical current presentations as predominantly inattentive, predominantly hyperactive/impulsive, or combined when symptoms of inattention or hyperactivity/impulsivity coexist [4]. In addition to the core symptoms, children/adolescents with ADHD have increased rates of comorbidities, including conduct disorder, learning disabilities, oppositional defiant disorder, or depressive disorders, that have further impact on the child/adolescent’s everyday life [5].

ADHD significantly impairs psychosocial functioning and adjustment, and children/adolescents usually have difficulties in interpersonal relationships with peers/family [6, 7]. Furthermore, poor academic performance is more common in children/adolescents with ADHD than in those without ADHD [8]. Consequently, compared with non-ADHD children/adolescents, children/adolescents with ADHD have low self-perception and self-esteem, and impaired quality of life [9–11]. Children/adolescents with ADHD have an increased risk of mortality, and school-age children with ADHD are also more likely to require emergency treatment or be admitted to hospital due to accidental injuries, whereas adolescents with ADHD have higher rates of early and more severe substance use, and of serious delinquent behaviors than their non-ADHD peers [12–15].

Health-economics literature has consistently shown that the overall financial burden of ADHD to children/adolescents, their families, and society is substantial [11, 16–18]. A study on costs of brain disorders in 30 European countries estimated that the direct annual costs of ADHD in 2010 were €781 per person, representing a total of €2546 million. These figures are lower than for autism (total of €15,109 million) and slightly lower than for conduct disorder (total of €3671 million) [19]. Moreover, ADHD annual health care costs per child/adolescent vary substantially across European countries (from €716 in Germany to €2134 in the Netherlands), and are of a similar order of magnitude in the US ($621 to $2720) [11]. However, comparisons are difficult regarding other types of costs; for instance, special educational support may be provided within mainstream schools in some countries (e.g. the UK), but are completely external in others (e.g., Spain).

Systematic reviews of the literature have shown that the pharmacological treatment of ADHD is associated with a substantial benefit in outcomes in the short term, and is cost-effective compared with placebo, no treatment, or behavioral therapy only [20, 21]. Analyses on the effect of treatment on long-term outcomes are scarce, but it has also been reported that untreated individuals experience worse outcomes overall than those receiving any treatment [10, 22, 23]. Therefore, individuals who are untreated or inappropriately treated, or who do not respond adequately to treatment, may experience and/or contribute to a significant increase in the short- and long-term financial burden of the disease as a result of additional expenses that can be attributed to the health, social, educational, and justice systems. There are few studies on the economic impact of ADHD in Europe to date, and those available are not directly comparable with studies published in the US because of putative underlying variations in treatment approaches or public health policies [5].

The objective of this multicenter, cross-sectional study was to estimate the associated direct and indirect costs from both a health care system and a societal perspective over a 1-year time period in Spanish children/adolescents with ADHD. In addition, we assessed whether the characteristics and financial costs differed between children/adolescents responding well (good responders) versus those who responded poorly (poor responders) to currently available pharmacotherapies.

## Participants and methods

### Study design

This was a multicenter, cross-sectional, descriptive study in a sample of children/adolescents with ADHD in the context of the Spanish research project “Plan of Action in ADHD (PANDAH)”. A total of 321 children/adolescents were recruited between 1 December 2012 and 30 April 2013 from 15 health care centers (7 privately and 8 publicly funded) representative of the overall Spanish population.

### Study population

The study included children/adolescents with DSM-IV Text Revision (DSM-IV-TR) diagnostic criteria for ADHD, aged ≤18 years, and without intellectual disability (i.e. intelligence quotient ≥70), as estimated by the treating physician. Children/adolescents were excluded if they had one of the following conditions: a severe medical disease other than asthma (e.g. neoplasm, diabetes mellitus, Crohn’s disease), neurological disorders (e.g. epilepsy), severe psychiatric disorders (including psychosis, bipolar disorder, and pervasive developmental disorders), symptoms of major depression before the onset of ADHD, or ADHD symptoms during episodes of depression.

To minimize selection bias, child/adolescent psychiatry or pediatric neurology units initially recruited the first child/adolescent who attended on a given day who fulfilled study inclusion criteria. When additional children/adolescents attended on the same day, only those consecutive to the first one were enrolled.

### Sample size calculation

To detect a mean cost difference of €2000 between good responders and poor responders, and assuming a standard deviation (SD) of €5000 and €7000, respectively, we calculated that the total sample size needed was 350 children/adolescents (α = 0.05; β = 0.2). As the study was conducted in 15 different health care centers, each one should recruit between 20 and 30 children/adolescents.

### Ethical considerations

The central Ethics Committee that approved the study was the Niño Jesús Hospital Committee (Madrid), and all children/adolescents or their legal representatives signed a written informed consent form prior to participation in the study.

### Data collection

A case report form (CRF) to be completed by the investigators was specifically designed for the study. First, a pilot study was conducted at 3 of the participating centers and data entered for 2 children/adolescents; this served to detect possible misleading questions, or other problems encountered by the investigators when completing the questionnaires. Second, all participating investigators were trained in the usage of the final CRF through a conference call. The CRFs were collected each week from all participating centers to build a common database.

### Study variables

At the time of recruitment, the following data were collected and recorded by the investigators during the interview: a) current demographic characteristics of children/adolescents; b) socio-occupational status; c) social relationships; d) date of diagnosis, ADHD subtype, and psychiatric comorbidities based on DSM-IV-TR criteria [24]; e) ADHD symptom severity for the last 6 months (baseline or current severity) using ADHD rating scale-IV (ADHD-RS-IV) [25]; f) severity of the disease at the time of recruitment evaluated using the Clinical Global Impression questionnaire [26]; g) general current functioning of the child/adolescent using the Children’s Global Assessment Scale (CGAS) [27]; h) the lowest known CGAS score during the previous year; and i) pharmacological and non-pharmacological ADHD-related treatments received in the previous 12 months. Available drugs for ADHD treatment in Spain at the time of the study included methylphenidate (MPH) OROS, MPH pellets, MPH immediate release (IR), and atomoxetine. Finally, the parents/caregivers were asked to provide data on utilization of health care services by the child/adolescent for the prior 12 months and their own work productivity, assessed using the Work Productivity and Activity Impairment questionnaire [28].

### Data analyses and statistics

#### Descriptive analysis

Descriptive analyses were summarized by means (SDs) or medians and interquartile ranges for continuous variables, and absolute numbers and percentages for categorical variables.

#### *Cost analysis*

The study was performed using a health care system and a societal perspective to estimate both direct and indirect costs, and adopted a 1-year time period.

All costs are expressed in 2012 Euros. Direct medical costs were defined as the cost of resources consumed to detect and treat the disorder, including costs of diagnostic tests, blood tests, emergency/outpatient visits, hospital admissions, and medication use. Direct medical costs were estimated by multiplying the amount of consumed resources by unit costs. These were obtained from the individual official reimbursed process in each particular region because, in Spain, health care is a competence of each autonomous community. Transport to treatment center was classified as a non-medical direct cost, and estimated based on public transport or taxi tickets given to the child/adolescent and parent/caregiver. Total direct costs were the sum of medical and non-medical costs. Indirect costs were those faced because of ADHD symptoms, and defined as the costs of the child/adolescent’s parents or caregivers, and the cost of lost productivity – as a consequence of hours spent in medical visits or missing workdays due to sick leave – applied to parents or caregivers. The cost of lost productivity was calculated by multiplying the estimated number of lost working hours by the average wage per working-hour [29]. More intangible costs, such as grade repetition, school drop-out, and time spent attending psychologist/educational psychologist visits, were described as proportions or means (SDs) (e.g. mean [SD] number of visits per year).

#### Comparison between good responders and poor responders

Good responders were defined as children/adolescents treated pharmacologically for at least 9 months who had an ADHD-RS-IV total score ≤ 18 (i.e. never, rarely, or sometimes ill) for at least the 3 previous months [30, 31]. Conversely, poor responders were children/adolescents who, despite receiving pharmacological treatment for at least 9 months, had an ADHD-RS-IV total score > 18. The comparison between good responders and poor responders was calculated using the chi-square or Fisher’s exact test for categorical variables. For continuous variables, the Student’s t test was used for normally distributed variables, and the Mann-Whitney test for non-normally distributed variables. *P*-values were unadjusted for multiplicity.

## Results

### Sample description and demographic characteristics

A total of 321 children/adolescents were enrolled in the study; 262 were recruited by child/adolescent psychiatrists, 59 by neuropediatricians. Table 1 summarizes the clinical and demographic baseline characteristics of the study sample. The age of the sample ranged between 6.3 and 18.0 years (mean [SD] 12.7 [2.9] years); most children/adolescents were male (78.5%) and 16 were younger than 8 years of age. The most frequent clinical presentation was combined ADHD type (61.4%), and the most common comorbidities were oppositional defiant disorder and anxiety disorders (19.9% and 17.8% of cases, respectively). At baseline, treating physicians rated children/adolescents as being mild to moderately ill in 73.9% of cases, and most had a good level of functioning with minor impairments (CGAS mean [SD] score = 74 [15.3]).

**Table 1:** Clinical and demographic baseline characteristics of the study sample (*N* = 321)

Characteristic	Cases
Age, years, mean (SD)	12.7 (2.9)
Age, years, range (P25, P75)	6.3–18.0 (10.6, 15.1)
Sex, n (%)	
*Male*	252 (78.5)
*Female*	69 (21.5)
ADHD presentation, n (%)	
*Combined*	197 (61.4)
*Predominantly inattentive*	112 (34.9)
*Predominantly hyperactive-impulsive*	12 (3.7)
ADHD-RS-IV score, mean (SD)	22.04 (10.3)
Duration of the disorder, years, mean (SD)	3.83 (2.5)
Current level of functioning (CGAS), mean (SD)	74 (15.3)
Lowest CGAS during the previous year, mean (SD)	56.1 (15.6)
Comorbidities, n (%)	
*Oppositional defiant disorder*	64 (19.9)
*Other conduct disorders*	19 (5.9)
*Anxiety disorders*	57 (17.8)
*Addictions*	6 (1.9)

*ADHD* attention-deficit/hyperactivity disorder; *ADHD-RS-IV* ADHD rating scale IV; *CGAS* Children’s Global Assessment Scale; *P25* 25th percentile; *P75* 75th percentile; *SD* standard deviation

Most children/adolescents were currently living with their parents and siblings (63.6%); their relationship with their family was good in 53.0% of cases. However, because of the ADHD symptoms, 17.8% of parents had to give up their job to care for their child/adolescent, and 11.5% of children/adolescents had a caregiver other than their parents, of whom 54% were relatives.

Relationship with peers was good overall (59.5% of cases), but, in 43.0% of cases, parents reported that the child/adolescent had some type of social difficulty such as being led by others, provoking and annoying others, or being sore losers (e.g. becoming very upset or angry when he or she loses a game, contest, etc).

All children/adolescents were attending school except one who was unemployed; 51% were enrolled in partially State-funded institutions, 35% in public schools, and 14% in private institutions. At the academic level, 19.1% of children/adolescents required an adapted school curriculum, and 51.9% received reinforcement/tutoring lessons about 4 times/week (mean [SD] = 4.2 [3.8]). A total of 13.7% of children/adolescents had been expelled from school at least once, with a mean (SD) age at first expulsion of 12 (2.5) years, and a mean (SD) of 3.3 (4) expulsions. Finally, 33.6% repeated a grade (mean [SD] repetitions = 1.1 [0.3]), at a mean (SD) age of 10.6 (2.8) years for the first time.

### **Cost analysis**

The average estimated total cost of ADHD per year per child/adolescent was €5732.64, with direct costs accounting for 60.2% of total costs (€3450.04) (Table 2).

**Table 2:** Estimates of annual resource use and costs in 321 children and adolescents with ADHD

Costs		Use of resource	Mean cost in € (SD)	Percentage of total cost
Direct medical costs	Visits to health services, mean (SD); median (P25, P75)		703.40 (785.64)	12.3%
	* Primary care center*	3.3 (5.8); 2 (0, 4)		
	* Psychiatrist*	5.02 (6.0); 4 (2, 6)		
	* Another specialist* ^a^	1.59 (3.5); 0 (0, 2)		
	Hospital admissions, mean (SD)		95.42 (958.49)	1.7%
	* Visits to emergency departments*	0.3 (0.8)		
	* Number of admissions*	0.01 (0.1)		
	* Days admitted*	0.02 (0.2)		
	* Stays in day hospital*	0.54 (6.0)		
	Diagnostic tests, mean (SD)		98.43 (205.46)	1.7%
	* Hemogram*	0.46 (0.8)		
	* Clinical chemistry*	0.47 (0.9)		
	* Urinalysis*	0.16 (0.7)		
	* NMR*	0.08 (0.4)		
	* EEG*	0.09 (0.3)		
	* ECG*	0.2 (0.4)		
	* CT scan*	0.01 (0.1)		
	* Evoked potentials*	0.01 (0.1)		
	Pharmacotherapy,^b^ n (%)		889.77 (674.72)	15.5%
	* MPH OROS*	210 (65.4)		
	* MPH pellets*	72 (22.4)		
	* MPH IR*	82 (25.5)		
	* Atomoxetine*	59 (18.4)		
	* Clonidine*	3 (0.9)		
	* Other*	37 (11.5)		
	Non-pharmacological treatment, n (%)		1561.14 (2768.53)	27.2%
	* Educational psychologist*	120 (37.4)		
	* Psychologist*	105 (32.7)		
Direct non-medical costs	Transportation to treatment center,^c^ n (%)		101.88 (182.89)	1.8%
	* Walking*	27 (8.4)		
	* Private car*	223 (69.5)		
	* Taxi*	2 (0.6)		
	* Public transport*	68 (21.2)		
Total direct costs			3450.04 (3370.92)	60.2%
Indirect costs	Caregiving, hours/day, mean (SD)	4.6 (2.5)	1488.16 (5141.73)	26.0%
	Caregiving, days/week, mean (SD)	4.9 (1.7)		
	Sick leave, days, mean (SD)^d^		2.55 (45.7)	<1.0%
	* Total sick leave*	8.5 (2.12)		
	* Leave due to ADHD*	5 (7.1)		
	Time spent at medical visits^d^		791.89 (625)	13.8%
Total indirect costs			2282.60 (5391.28)	39.8%
Total costs			5732.64 (7211.39)	100%

^a^This includes neuropediatricians, but not psychologists/educational psychologists; ^b^Children/adolescents were allowed to have more than one pharmacological treatment; ^c^*n* = 320; ^d^Applied to parents or caregivers of children/adolescents with ADHD

*ADHD* attention-deficit/hyperactivity disorder; *CT* computerized tomography; *ECG* electrocardiogram; *EEG* electroencephalogram; *IR* immediate release; *P25* 25th percentile; *P75* 75th percentile; *MPH* methylphenidate; *NMR* nuclear magnetic resonance; *SD* standard deviation

The most frequently accessed health service was a visit to a psychiatrist, with a median of 4 visits per year (25th percentile [P25] = 2; 75th percentile [P75] = 6), followed by visits to a primary care center (median of 2 visits; P25 = 0; P75 = 4). Support from an educational psychologist or a psychologist (non-pharmacological treatment) was needed by 37.4% and 32.7% of children/adolescents, respectively, in the majority of cases held at private centers (75.8% and 62.8%, respectively), and represented 45.2% of direct costs and 27.2% of total costs. Pharmacotherapy accounted for 25.8% of direct costs and 15.5% of total costs. Stimulants, specifically MPH OROS (65.4%) and MPH IR (25.5%) were the most commonly prescribed drugs. The mean duration of treatments during the previous year was 288 and 252 days for MPH OROS and MPH IR, respectively (data not shown). Costs due to hospital admissions, diagnostic tests, and transportation to treatment centers represented 2% of total costs each.

**Fig. 1:**
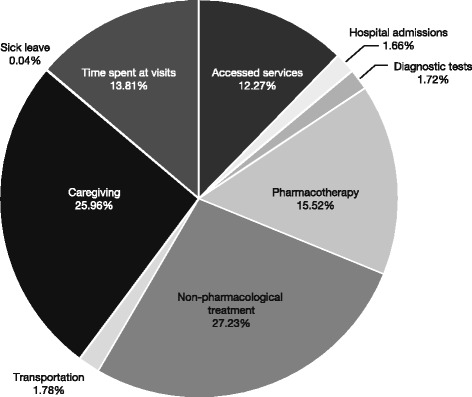
National ADHD-related costs by cost categories

Among the indirect costs estimated, 65.2% was due to caregiver expenses, which were paid by a relative in 35.1% of cases (data not shown), and represented 26% of the total costs. Moreover, a parent or caregiver had to leave their job to care for the child/adolescent in 17.8% of cases, and the average number of lost working days in the previous year was 8.5, of which 5 (SD 7.1) days were due to ADHD itself (Fig. 1 and Table 2).

### Impact of response to ADHD treatment

In the overall sample, 73.5% (*n* = 236) of children/adolescents received pharmacological treatment, and treating physicians considered that 58.8% (*n* = 139) were good responders.

There were no differences between good and poor responders with respect to type of treatment or treatment duration, but good responders (*n* = 139) had less severe ADHD measured at baseline, greater improvement from baseline, and were less frequently diagnosed with oppositional defiant disorder (Table 3 and Additional file 1: Table S1). Moreover, in poor responders (*n* = 97), the disorder had a more negative impact on several aspects of their lives, including poorer global functioning, with worse relationships with friends and family.

**Table 3:** Significant differences between children/adolescents with good response versus those with poor response to pharmacological treatment

	Good responders (*n* = 139)	Poor responders (*n* = 97)	*P*-value
Characteristics of the disease, mean (SD)			
*Current level of functioning (CGAS)*	78.6 (12.9)	67.9 (16.3)	<0.0001
*Lowest CGAS score during the previous year*	58.5 (14.2)	67.9 (16.3)	0.001
Current severity, n (%)			
*Moderately-severely ill*	36 (25.9)	63 (64.9)	<0.0001
Comorbidities, n (%)			
*Oppositional defiant disorder*	20 (14.4)	27 (27.8)	0.01
*Other conduct disorders*	7 (5.0)	8 (8.2)	0.40
*Anxiety*	21 (15.1)	24 (24.7)	0.07
*Addictions*	1 (0.7)	4 (4.1)	0.16
Impact on social functioning, n (%)			
*Bad relationship with friends*	1 (0.7)	15 (16.7)^a^	<0.0001
*Bad relationship with family*	7 (5.0)	22 (22.7)	<0.0001

^a^*n* = 90

*CGAS* Children’s Global Assessment Scale, *SD* standard deviation

**Fig. 2:**
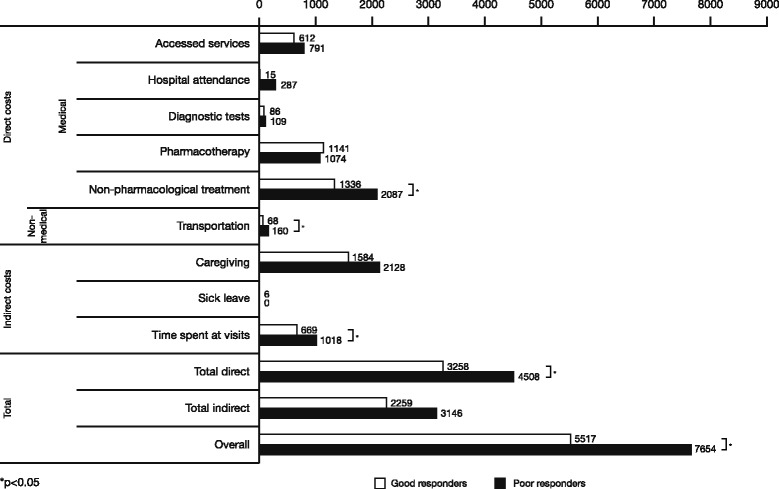
ADHD costs (€) in children/adolescents with adequate pharmacological treatment response versus those for whom treatment failed

The difference in total annual costs of ADHD between groups was statistically significant, and higher for poor responders compared with good responders (mean [SD]: €7654 [€8966] versus €5517 [€6618]; *P* = 0.024). This was mainly due to significantly higher direct costs for poor responders versus good responders (mean [SD]: €4508 [€3922] versus €3258 [€2635]; *P* = 0.005), attributable to higher expenses in non-pharmacological treatment and transport (*P* = 0.012 and <0.0001, respectively) (Fig. 2). Finally, there was a trend towards a greater economic impact on indirect costs for poor responders (*P* = 0.058) driven by significantly longer time spent at visits (*P* = 0.011).

## Discussion

In this study, we evaluated the economic impact of ADHD on the utilization of health care resources and societal costs in Europe as well as the costs of this neurodevelopmental disorder in children/adolescents from the societal perspective. In addition, we assessed how these costs differed between children/adolescents who adequately responded to pharmacological treatment compared with those for whom treatment response was poor.

Although few studies have assessed the economic impact of ADHD on the utilization of health care resources and societal costs in Europe, estimates of costs vary between studies depending on types of services included and methodology. The annual health care costs per child/adolescent with ADHD in Europe (adjusted to 2012 Euros) have been estimated to be between €716 and €3888 [11, 32]. The results of this study are in line with these estimates, with direct medical costs in Spain of €3450.04, accounting for 60.2% of all expenditures. However, it is largely recognized that, unlike many medical diseases, a significant proportion of the financial burden of psychiatric illnesses also arises from indirect costs [33]. The few available studies conducted in the European Union that estimated indirect costs of ADHD found values varying between €50 and €5596 per year, a difference that may be largely explained by the type of services considered [11, 34–36]. In the current study, indirect costs amounted to €2282.60, and were driven mainly by payment to a caregiver.

The main contributor to direct costs corresponded to non-pharmacological interventions (e.g. intervention by an educational psychologist or clinical psychologist), and accounted for 45.2% of total direct costs and 27.2% of total costs. Most cost-of-illness studies in ADHD include these services together with costs of other health care professionals, and therefore the specific weight of costs due to psychological interventions is difficult to compare. Nonetheless, these results are similar to a German study that considered the costs of behavioral therapy separately from other types of interventions, and reported that this expenditure represented 46.6% of costs for children/adolescents aged 6–17 years, and up to 74.4% in children ≤5 years of age [32]. Moreover, we did not consider special education needs, which might have contributed to an underestimation of the total costs in our study. Available European studies (conducted in the UK) that considered the costs of educational needs, such as special school, support teacher, or adapted school curriculum, reported them to be about 3-fold the sum of all other costs [36], or 44% of the total costs [18], and they were estimated to account for 30–40% of all expenditures in the US [16]. Although not directly comparable, our results seem to be in line with these estimates of the high burden due to special educational needs in children/adolescents with ADHD. The average proportion of costs of medication in the present study was 15.5%, which is in line with previous European studies that estimated that they range between 12% and 28.2% [32, 37–39].

One striking finding of our study was the high cost associated with the employment of a caregiver, which represented 65.2% of indirect costs, and 26% of all costs. Mental health-related problems in caregivers and families of children/adolescents with ADHD include stress, anxiety, and depression [40], but the financial burden on caregivers has not been routinely explored [1]. In our study, a parent had to leave their job to care for the child/adolescent in 17.8% of cases, thus hiding a significant unquantified productivity loss. Moreover, this shows that the costs we estimated to caregiving, although high, are largely an underestimation of the actual economic impact on families and society as a whole.

In this study, poor responders were observed to have worse global functioning, and poorer relationships with friends and family. This is in line with the results of several systematic reviews, which reported that treatment has a beneficial effect on multiple short- and long-term outcomes, including self-esteem, social functioning, academic performance, and antisocial behavior, among others, when compared with children/adolescents without ADHD or those with untreated ADHD [10, 22, 23]. Our results, however, also show that beyond symptom control, a poor response to treatment is an important component in the global negative impact of ADHD. This stresses the need for individualized treatment plans to optimize child/adolescent’s outcomes, such as the incorporation of patient-oriented factors (e.g. age, ADHD subtype, comorbidities, and treatment/treatment response history) as well as the preferences and attitudes of children/adolescents and parents/caregivers to pharmacotherapy [41].

Poor responders were found to incur higher total financial expenses than good responders, a difference mainly due to direct medical costs, and particularly because of significantly higher costs of non-pharmacological treatment. Although not directly comparable, these results are in line with previous systematic reviews showing that treatment is associated with less use of ancillary services, including school-, health-, and work-related services; financial assistance services; use of the justice system; and fewer emergency room visits [22, 23].

The main advantage of the current study is that the studied population is representative of different regions in Spain and data were complete for almost all variables studied. However, the results must be interpreted in the light of considerable limitations. First, the cross-sectional nature of the study, with information obtained retrospectively through interview for some variables, means that results may be more sensitive to potential recall bias. Second, children/adolescents recruited in this study were treated by psychiatrists and neuropediatricians, and may therefore be representative of a subset of children/adolescents with more severe ADHD, thus incurring higher costs, as for adults [42]. Third, there are several factors that were not quantified in the study that might have contributed to an underestimation of the total ADHD-related costs. For example, we did not include additional indirect costs such as health-related problems among family members, although these may be considerable too, as suggested by a Dutch study that estimated annual average health care costs per family member as 14.3% of all health care costs [11, 37]. Moreover, we did not estimate costs associated with psychiatric/non-psychiatric comorbidities, which have been shown to cause a 6-fold increase in medical costs compared with ‘pure’ ADHD cases [37]. Although excluding comorbidities can lead to a significant underestimation of the total cost of illness, when it is difficult to separately assess the expenditures of closely related disorders, it can also potentially lead to an inflation of cost estimates [43]. In addition, we did not estimate ADHD-related costs due to the justice system, treatment of comorbid substance abuse, or traffic accidents. None of the available European studies have quantified these totals, but the costs of justice system service needs for children/adolescents with ADHD in the US were estimated to be about 1% of the total costs of ADHD [16].

## Conclusions

ADHD is a highly prevalent disorder associated with a significant high personal, familial, and financial impact on the family, health system, and society. An adequate response to pharmacological treatment was associated with lower overall expenses in the management of the disorder.

## Additional file

Additional file 1: Table S1. Non-significant differences in clinical and demographic baseline characteristics between children/adolescents with good response versus those with poor response to pharmacological treatment. The table presents analysis results and is provided as a supplement to Table 3 Significant differences between children/adolescents with good response versus those with poor response to pharmacological treatment. (DOCX 23 kb)

## Abbreviations

ADHD: Attention-deficit/hyperactivity disorder
ADHD-RS-IV: Attention-deficit/hyperactivity disorder rating scale, version IV
CGAS: Children’s Global Assessment Scale
CRF: Case report form
DSM-5: Diagnostic and Statistical Manual of Mental Disorders, Fifth edition
DSM-IV-TR: Diagnostic and Statistical Manual of Mental Disorders, Fourth edition, Text Revision
IR: Immediate release
MPH: Methylphenidate
P25: 25th percentile
P75: 75th percentile
SD: Standard deviation
