# Supplementary material for: Health care and societal costs of the management of children and adolescents with attention-deficit/hyperactivity disorder in Spain: a descriptive analysis
Source: BMC Psychiatry. 2018 Feb 8;18:40. doi: 10.1186/s12888-017-1581-y (PMC5806309; doi:10.1186/s12888-017-1581-y)
Supplement: Supplementary file 1 — Non-significant differences in clinical and demographic baseline characteristics between children/adolescents with good response versus those with poor response to pharmacological treatment. The table presents analysis results and is provided as a supplement to Table 3 Significant differences between children/adolescents with good response versus those with poor response to pharmacological treatment. (DOCX 23 kb) [file 12888_2017_1581_MOESM1_ESM.docx]

## Supplementary Table 1 Non-significant differences in clinical and demographic baseline characteristics between children/adolescents with good response versus those with poor response to pharmacological treatment

|  | Good responders  (*n* = 139) | Poor  responders  (*n* = 97) | *P*-value |
| --- | --- | --- | --- |
| Age, years, mean (SD) | 13.1 (2.9) | 13.0 (2.6) | 0.82 |
| Sex, n (%)  *Male*  *Female* | 112 (80.6)  27 (19.4) | 76 (78.4)  21 (21.6) | 0.74 |
| ADHD presentation, n (%)  *Combined*  *Predominantly inattentive*  *Predominantly hyperactive-impulsive* | 81 (58.3)  54 (38.9)  4 (2.9) | 65 (67.0)  28 (28.9)  4 (4.1) | 0.28 |
| Duration of the disorder, years, mean (SD) | 3.7 (2.3) | 4.0 (2.6) | 0.54 |
| Pharmacotherapy, n (%)  *MPH OROS*  *MPH pellets*  *MPH IR*  *Atomoxetine*  *Clonidine*  *Other* | 93 (66.9)  32 (23.0)  31 (22.3)  28 (20.1)  0 (0.0)  7 (5.0) | 60 (61.9)  21 (21.7)  16 (16.5)  20 (20.6)  3 (3.1)  21 (21.7) | 0.49  0.88  0.32  1.0  0.07  NA |
| Treatment duration, days, mean (SD)  *MPH OROS*  *MPH pellets*  *MPH IR*  *Atomoxetine*  *Other* | 339.6 (64.9)  304.6 (94.5)  262.9 (140.2)  252.3 (139.1)  301.4 (132.8) | 324.3 (78.3)  278.9 (125.3)  342.8 (58.6)  274.3 (107.5)  225.0 (140.0) | 0.11  0.42  0.16  0.66  0.22 |
| Non-pharmacological treatment  *Educational psychologist, n (%)*  *Treatment duration, days, mean (SD)*  *Psychologist, n (%)*  *Treatment duration, days, mean (SD)* | 47 (33.8)  38.3 (39.4)  39 (28.1)  16.2 (15.5) | 41 (42.3)  46.7 (47.0)  34 (35.1)  21.4 (14.7) | 0.22  0.21  0.32  0.09 |

*ADHD* attention-deficit/hyperactivity disorder*; IR* immediate release; *MPH* methylphenidate; *NA* not applicable; *SD* standard deviation
